# Effects of Preoperative Oral Carbohydrate on Perioperative Maternal Outcomes Undergoing Cesarean Section: A Systematic Review and Meta-Analysis

**DOI:** 10.1155/2024/4660422

**Published:** 2024-03-31

**Authors:** Haibin Shi, Caihong Zheng, Bin Zhu

**Affiliations:** ^1^Department of Anesthesiology, Affiliated Hangzhou First People's Hospital, School of Medicine, Westlake University, Hangzhou, Zhejiang, China; ^2^Department of Anesthesiology, Hangzhou Women's Hospital, Hangzhou, Zhejiang, China

## Abstract

**Purpose:**

Preoperative oral carbohydrate (CHO) is a rapid postoperative rehabilitation protocol that improves perioperative outcomes and is widely used in adult surgical patients. However, pregnant women are excluded because of the possibility of aspiration due to delayed gastric emptying. This meta-analysis was conducted to evaluate the efficacy of preoperative oral CHO in elective cesarean section.

**Methods:**

PubMed, Embase, Web of Science, and the Cochrane Library were searched from inception to July 2023. Randomized controlled trials were included. The risk of bias was assessed using the Cochrane tool. Risk ratios and 95% confidence intervals were calculated. Meta-analysis was performed using random-effects models to estimate risk ratios and mean differences (MDs) with 95% confidence intervals (CIs). The outcomes included thirst and hunger scores, incidence of vomiting and nausea, time to flatus, and homeostatic model assessment of insulin resistance (HOMA-IR).

**Results:**

A total of nine studies with 1211 patients were included in the analysis. The levels of thirst and hunger were evaluated using a 10-point visual analog scale, with 0 representing the best and 10 representing the worst. The severity of hunger (weighted mean difference (WMD: −2.34, 95% CI: −3.13 to −1.54), time to flatus (WMD: −3.51 hours, 95% CI: −6.85 to −0.17), and HOMA-IR (WMD: −1.04, 95% CI: −1.31 to −0.77) were significantly lower in the CHO group compared to the control group. However, there were no significant differences in the severity of thirst or the incidence of vomiting and nausea between the CHO and control groups.

**Conclusion:**

Preoperative oral CHO during cesarean section alleviates thirst and hunger, shortens the time of postoperative flatus, and reduces HOMA-IR. However, the available evidence is insufficient to reach a clear consensus on the benefits or harms of preoperative oral CHO during cesarean section. Therefore, it is premature to make a definitive recommendation for or against its routine use.

## 1. Introduction

Pulmonary aspiration of gastric contents is a rare but potentially life-threatening complication, with 57% of aspiration events resulting in death and another 14% resulting in permanent serious injury [1]. Pregnancy increases the risk of pulmonary aspiration during anesthesia and surgery due to decreased lower esophageal sphincter tone and delayed gastric emptying, so preoperative fasting has been recommended to minimize gastric contents [2, 3]. However, prolonged fasting before surgery may have adverse effects such as hunger, thirst, dry mouth, fatigue, anxiety, and postoperative insulin resistance [4].

Enhanced recovery after surgery (ERAS) is a multimodal perioperative care pathway designed to achieve early recovery for patients undergoing major surgery and has been widely adopted in various settings [5–7]. Shortening preoperative fasting time and preoperative oral carbohydrate consumption are the core recommendations of ERAS for patients undergoing elective surgery [8]. Preoperative administration of oral carbohydrates is recognized for its ability to reduce metabolic stress and insulin resistance after surgery, leading to shorter hospital stays and improved well-being in patients undergoing abdominal, orthopedic, and cardiac surgery [9–12]. However, compared to the practice of fasting at midnight, preoperative oral carbohydrate intake does not appear to improve patient satisfaction or well-being in individuals undergoing thyroidectomy [13].

Recently, several randomized controlled trials (RCTs) have shown that patients undergoing elective cesarean section who consumed carbohydrate solutions had fewer postoperative side effects and complications than those who fasted or received a placebo [14–21]. This approach has also been shown to be safe and feasible in patients with gestational diabetes mellitus (GDM) [22]. In addition, both a standard oral rehydration solution and a high-carbohydrate drink consumed prior to surgery have been shown to provide superior comfort compared to fasting [23]. However, it is important to note that the sample size of each study was relatively small, limiting the reliability of the results, and no meta-analysis has been performed. In order to assess the impact and safety of preoperative oral carbohydrate intake in pregnant women who are scheduled for cesarean section, we decided to conduct an extensive systematic review and meta-analysis.

## 2. Material and Methods

In this study, we followed the guidelines set forth in the Preferred Reporting Items for Systematic Reviews and Meta-Analyses (PRISMA) statement [24]. This ensures that our research is conducted and reported in a transparent and comprehensive manner. Moreover, it was duly registered with the International Prospective Register of Systematic Reviews under the registration number CRD42020177211.

### 2.1. Search Strategy

A methodical screening approach was implemented to identify pertinent literature in PubMed, Web of Science, Embase, and the Cochrane Library from the time of their establishment until July 2023. The exploration strategy encompassed Medical Subject Headings (MeSH) terms: “carbohydrates,” “dietary carbohydrates,” “cesarean section,” and “randomized controlled trial.” Supplementary Appendix A provided additional terms for inclusion. The National Library of Medicine's primary screening queries were monitored on a weekly basis until September 1, 2023, but no relevant findings were discovered. Bibliographies of chosen articles were also assessed to identify eligible trials.

### 2.2. Study Selection Criteria

This review included peer-reviewed RCTs, with or without blinding. Inclusion criteria were as follows: (1) pregnant women undergoing elective cesarean section, (2) oral carbohydrate intervention before surgery, (3) one or more of the following outcomes: thirst, hunger, nausea, vomiting, HOMA-IR (homeostatic model assessment for insulin resistance), and time to flatus, and (4) RCT design. Exclusion criteria were (1) studies with missing data. No RCTs were excluded based on factors such as the definition of intervention allocation or primary and secondary outcomes.

### 2.3. Data Extraction

Before the review process began, an independent reviewer (BZ) prepared a standardized data extraction form. Following that, the required data from the included articles were independently extracted by two authors (HBS and CHZ). This extracted data included information such as the names of authors, publication year, country, type of anesthesia, number of patients, intervention and comparison groups, and outcomes. In case of any conflicts during the data extraction process, a third author (BZ) resolved them.

### 2.4. Study Quality Assessment

Two authors (HBS and CHZ) independently assessed the selected studies using the Cochrane risk-of-bias tool to determine their risk of bias and methodological quality [25]. The risk of bias was evaluated across seven domains, including selective reporting, allocation concealment, blinding of participants and personnel, blinding of outcome assessment, incomplete outcome data, random sequence generation, and other biases. Each study was categorized as having a low, high, or unclear risk of bias. In cases of disagreement, consensus was reached by involving another author (BZ).

### 2.5. Statistical Analysis

Data processing was conducted using Review Manager Ver. 5.3 (Copenhagen, Nordic Cochrane Centre, Cochrane Collaboration, 2014). The Mantel–Haenszel test for random effects was used for data analysis. Risk ratios (RRs) with 95% confidence intervals (CIs) were reported for dichotomous outcomes. For continuous outcomes, weighted mean differences (WMDs) were reported. Statistical significance was defined as a *p* value of ≤0.05.

We used the *I*^2^ test to assess the heterogeneity of the articles. If *I*^2^ was <50% and *p* was >0.1, heterogeneity between studies was considered low. Insulin resistance was assessed using the homeostasis model assessment of insulin resistance (HOMA-IR), in which fasting blood insulin and glucose levels were measured according to the HOMA-IR formula. In cases where a randomized controlled trial (RCT) had an excess of two treatment arms, all pertinent information was incorporated. Subgroup meta-analyses were performed based on the control strategy used in the trials, which could be either placebo or fasting. Subgroup meta-analyses were conducted, taking into account the control strategy utilized in the trials, which might consist of either placebo or fasting.

## 3. Results

### 3.1. Search Results

**Figure 1:**
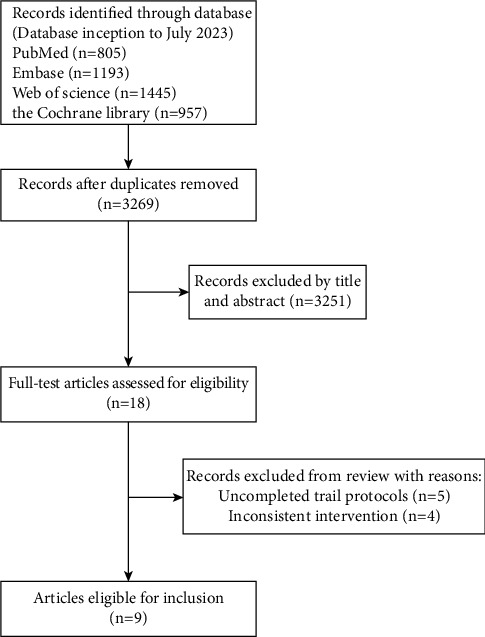
Flowchart of the literature search and selection of the included randomized controlled trials.

A total of 4400 papers were identified through a comprehensive search on various databases, including PubMed (*n* = 805), Web of Science (*n* = 1445), Embase (*n* = 1193), and the Cochrane Library (*n* = 957). After eliminating any duplicated papers, we proceeded to assess a total of 3269 articles for their suitability. Out of these, 3251 articles were excluded based on a thorough examination of their titles and abstracts. The remaining 18 studies underwent a detailed review of their full texts, resulting in the exclusion of 5 studies due to inconsistency and 4 studies due to intervention mismatch with our research. Ultimately, we identified 9 eligible randomized controlled trials (RCTs) for our analysis. The flow diagram illustrating the PRISMA study process can be found in Figure 1.

### 3.2. Study Characteristics

In Table 1, the details of the included RCTs are presented. These studies were published within the period of 2014 to 2022 and were conducted in four distinct countries. The sample sizes varied from 47 to 411 across all studies. In all studies, the intervention group received all CHO, while the control group received water [15–17, 19–22], 5.9% CHO [23], or fasting [15, 18–21, 23]. Outcomes included thirst and hunger scores, incidence of vomiting and nausea, time to flatus, and insulin resistance. One study [18] was administered under general anesthesia, and the other eight [15–17, 19–23] were administered under spinal anesthesia.

### 3.3. Risk-of-Bias Assessment

**Figure 2:**
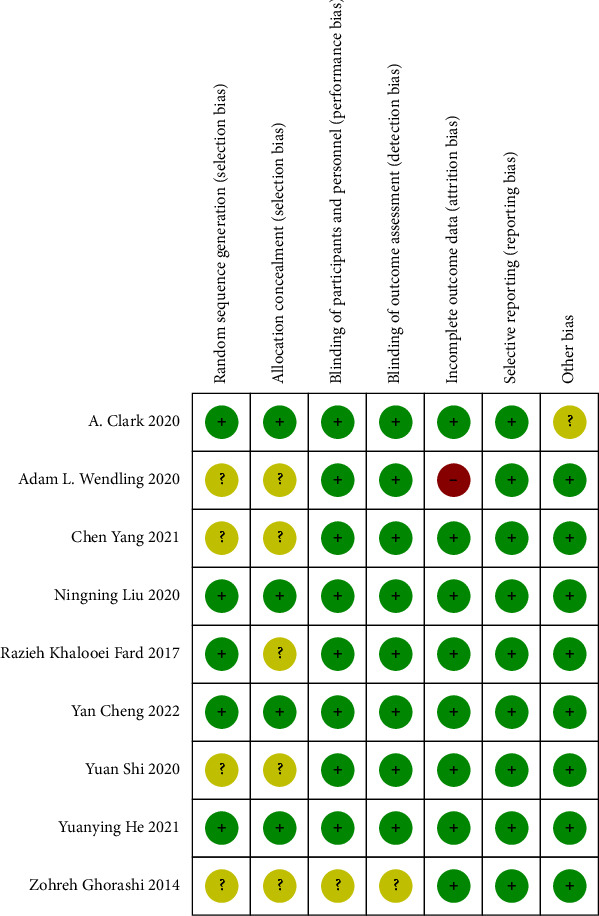
Risk-of-bias summary of the included studies. A green “+” indicates that the quality element was met, a red “−” indicates that the element was not met, and a yellow “?” indicates that it was uncertain whether the element was met.

The quality of the studies included in this analysis was evaluated using the Cochrane risk of bias tool. Out of the nine studies examined, eight [15–17, 19–23] of them were found to be double-blind, indicating a low risk of performance and detection bias. However, five studies [17, 18, 20, 21, 23] were considered with an unclear information on allocation concealment and random sequence generation. In addition, one study [23] excluded 40% of patients in the CHO group and 31.8% of patients in the fasting group due to loss to follow-up and discontinuation of the intervention, resulting in a high risk of attrition bias. The quality of a study increased along with the higher number of low risks (green pluses) of bias assessments. Another study [16] that used a sponsored carbohydrate was also considered with an unclear information.  Figure 2 presents the outcomes of the bias evaluation for each individual study.

### 3.4. Thirst

**Figure 3:**
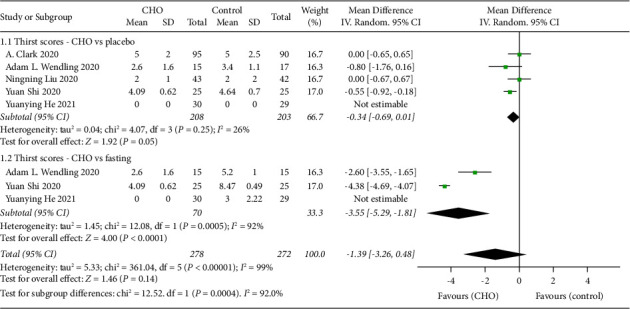
Forest plot showing the mean difference in thirst scores between CHO and control groups in randomized controlled trials of cesarean section.

Five studies [16, 19, 20, 22, 23] involving 480 patients compared the preoperative thirst levels between the groups receiving CHO and the control groups. Thirst levels were measured using the Visual Analog Scale (VAS), which ranges from 0 to 10, with 0 indicating no thirst and 10 indicating the highest level of thirst. Among the five control groups, four [16, 19, 20, 22] used water and one [23] used a low concentration of CHO. In addition to comparing CHO with water or low CHO concentration, three [19, 20, 23] of the studies also compared CHO with fasting. Subgroup analysis was performed according to whether the control group was placebo or fasting. In summary, the pooled results revealed no significant difference between CHO and placebo. However, it was observed that the CHO group showed a significant decrease in thirst levels, as compared to the fasting group (WMD: −3.55, 95% CI: −5.29 to −1.81) (Figure 3). Furthermore, one study [19] reported that preoperative oral CHO or water before surgery can quench thirst with a VAS score of 0.

### 3.5. Hunger

**Figure 4:**
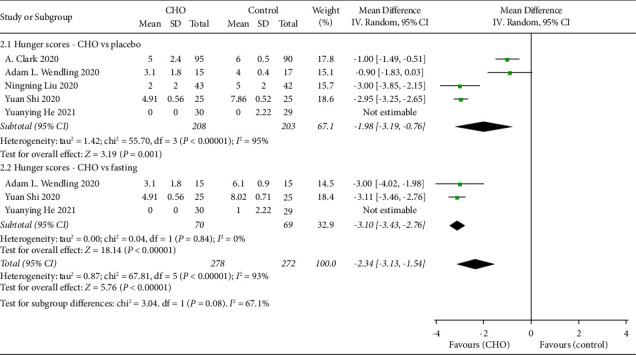
Forest plot showing the mean difference in hunger scores between CHO and control groups in randomized controlled trials of cesarean section.

The five studies [16, 19, 20, 22, 23] also compared preoperative hunger levels between the CHO and control groups using the VAS score (Figure 4). The pooled results indicated that preoperative oral CHO led to lower hunger scores compared to water or low concentration (WMD: −1.98, 95% CI: −3.19 to −0.76) and fasting (WMD: −3.10, 95% CI: −3.43 to −2.76). Overall, the findings demonstrated that preoperative oral CHO resulted in lower hunger levels compared to the control group (WMD: −2.34, 95% CI: −3.13 to −1.54).

### 3.6. Vomiting and Nausea

Three [15, 19, 21] studies involving a total of 319 patients were conducted to compare the incidence of vomiting between the CHO and control groups. The pooled result of these studies revealed that CHO did not have a significant impact on the incidence of vomiting compared to water (RR = 0.70; 95% CI: 0.28 to 1.77) or fasting (RR = 0.46; 95% CI: 0.20 to 1.09). The overall outcome indicated that preoperative oral CHO did not result in a significant difference in the incidence of vomiting compared to the control group (RR = 0.56; 95% CI: 0.30 to 1.05).

**Figure 5:**
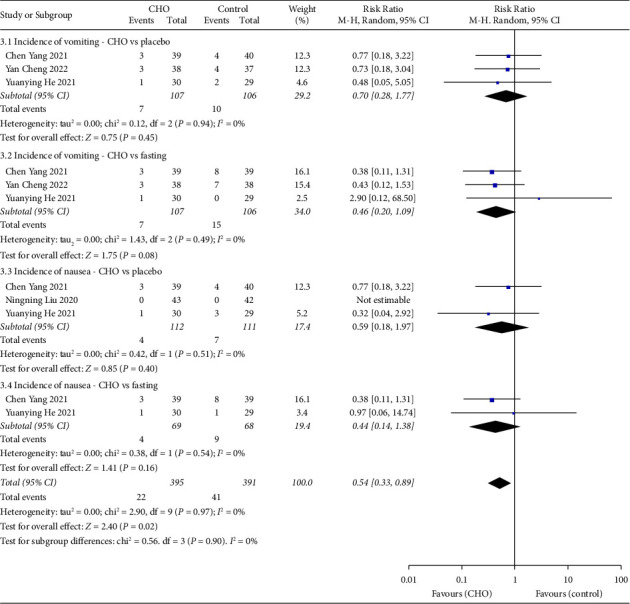
Forest plot showing the incidence of vomiting and nausea between the CHO and control groups.

Similarly, in three [19, 21, 22] studies involving 291 patients, the incidence of nausea was compared between the CHO and control groups. The pooled result for nausea revealed that CHO did not affect the incidence of nausea compared to water (RR = 0.59; 95% CI: 0.18 to 1.97) or fasting (RR = 0.44; 95% CI: 0.14 to 1.38). The overall outcome indicated that preoperative oral CHO did not result in a significant difference in the incidence of nausea compared to the control group (RR = 0.51; 95% CI: 0.22 to 1.16) (Figure 5).

### 3.7. Time to Flatus

**Figure 6:**
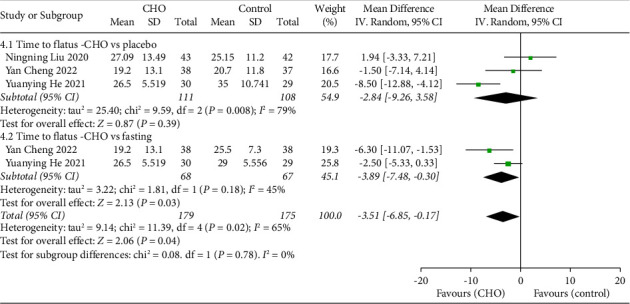
Forest plot showing the mean difference in time to flatus after cesarean section between CHO and control groups.

Three studies [15, 19, 22] involving 286 patients compared the time to return of flatus after cesarean section between the CHO and control groups. The pooled result indicated that CHO did not significantly alter the time to flatus compared to water (WMD: −2.84 hours, 95% CI: −9.26 to 3.58). However, the pooled result demonstrated a significantly shorter time to flatus in the CHO group compared to the fasting group (WMD: −3.89, 95% CI: −7.48 to −0.30). Overall, the findings demonstrated that preoperative oral CHO led to a shorter time to flatus compared to the control group (WMD: −3.51, 95% CI: −6.85 to −0.17) (Figure 6).

### 3.8. Insulin Resistance

**Figure 7:**
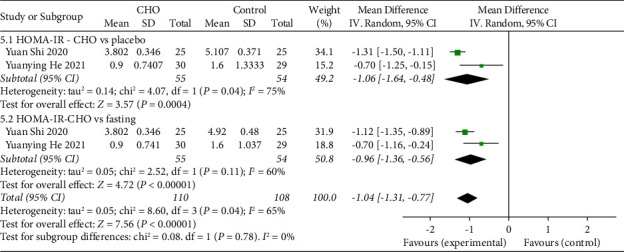
Forest plot showing insulin resistance between CHO and control with HOMA-IR.

Two studies [19, 20], involving a total of 163 patients, compared insulin resistance between the CHO group and the control group using HOMA-IR. The collective finding indicated that preoperative oral CHO intake yielded diminished HOMA-IR levels in contrast to either water ingestion (WMD: −1.06, 95% CI: −1.64 to −0.48) or fasting (WMD: −0.96, 95% CI: −1.36 to −0.56). The comprehensive outcome substantiated that the consumption of oral CHO before surgery led to considerably inferior HOMA-IR levels when compared to the control group (WMD: −1.04, 95% CI: −1.31 to −0.77) (Figure 7).

### 3.9. Other Outcomes

#### 3.9.1. Aspiration

A total of two studies [18, 23] involving 458 patients reported the incidence of aspiration in CHO groups and controls. In one study [18], the CHO group received 150 mL of 10% CHO orally 1 hour before induction of general anesthesia, while the control group was fasted. In another study [23], the CHO group received 355 mL of 14% CHO orally 2 to 4 hours before induction of spinal anesthesia, while the control group received 355 mL of 5.9% CHO or fasted. Also, no patient in any group experienced gastric aspiration.

#### 3.9.2. Time to Colostrum

Two studies [15, 17] involving 203 patients reported the time to colostrum in CHO groups and controls. One study [17] reported the time to colostrum after surgery was significantly shorter in the CHO group than in the water group (27.47 ± 11.51 vs. 51.96 ± 20.20 min, *p* < 0.001). Another study [15] reported that the time to colostrum was significantly shorter in the CHO group than in the water or fasting group (20.4 ± 13.7 vs. 39.8 ± 18.8 or 41.3 ± 17.6 h, respectively, *p* < 0.001).

#### 3.9.3. Apgar Score

Two studies [19, 23] involving 135 patients, respectively, reported Apgar scores at 1 and 5 minutes after birth. One study [23] showed that there was no Apgar score less than 7 at 5 minutes after birth. Also, another study [19] showed that the 1-minute Apgar scores were all 10 in both the CHO and control groups.

## 4. Discussion

This meta-analysis consisted of nine RCTs that systematically evaluated the effects of oral carbohydrate intake on perioperative maternal outcomes following cesarean section. Preoperative oral CHO undergoing cesarean section significantly alleviated thirst and hunger, shortened time to flatus and colostrum, and reduced postoperative insulin resistance compared with the fasting group, while there was no difference in the incidence of nausea, vomiting, and aspiration. In addition, oral CHO could significantly alleviate hunger and reduce postoperative insulin resistance compared with the water group. Therefore, oral administration of CHO before cesarean section appears to be a feasible procedure to minimize patient discomfort without additional risk.

Preoperative fasting has become a standard procedure due to the risk of anesthesia-related aspiration [26–28]. However, fasting can lead to patient discomfort and increase insulin resistance. [29]. One meta-analysis found that preoperative CHO loading may reduce patient discomfort in several elective procedures, including cesarean delivery, without safety concerns [30]. Conversely, another meta-analysis concluded that there is insufficient evidence to support the claim that preoperative CHO administration reduces patient discomfort. Nevertheless, preoperative CHO loading has been associated with postoperative insulin resistance and the incidence of postoperative infection [31]. Our review included nine RCTs with 1211 participants. Two [18, 23] of these RCTs with 458 patients reported the incidence of aspiration in both the CHO groups and the control groups undergoing cesarean section. With oral 150 mL of 10% CHO 1 hour before induction of general anesthesia or oral 355 mL of 14% CHO 2 to 4 hours before induction of spinal anesthesia, no patient in either group experienced gastric aspiration. Two [19, 20] studies involving 163 patients showed that preoperative oral CHO reduced insulin resistance compared with controls. Preoperative administration of CHO may minimize patient discomfort and did not increase the risk of aspiration.

The effects of this intervention may be influenced by the inclusion of nine studies with varying amounts and concentrations of CHO consumed. The concentrations of CHO used in the nine studies varied from 5.9% to 14.2%, and patients were given 300 to 400 mL of these CHO 2 hours before surgery. This was found to have no adverse effect on patient safety compared with fasting after midnight. In addition, a study [18] of 411 patients found that ingestion of 150 mL of a 10% CHO solution 1 hour before surgery did not result in aspiration in patients under general anesthesia. Therefore, a regimen of 300 to 400 mL of 5.9% to 14.2% CHO 2 hours before anesthesia is safely recommended instead of fasting after midnight the night before surgery. In addition, a study [23] comparing 14% and 5.9% CHO showed that the higher concentration of CHO improved hunger. However, either a low concentration rehydration drink or a high concentration of CHO consumed preoperatively resulted in superior comfort compared to fasting. Two studies [15, 17] compared the time to colostrum after surgery and found that the OCH group had a significantly shorter time compared to the water or fasting groups. It is worth noting that there is a significant difference in the mean time between the two groups, which may be due to racial differences, and more research is needed to confirm this.

The study has some limitations that should be considered. First, it is important to note that the methods of anesthesia used in the included studies varied. While eight studies utilized intraspinal anesthesia, only one study used general anesthesia. This difference in anesthesia methods may potentially affect the incidence of aspiration. Second, there is a paucity of studies examining various observational indicators, such as colostrum secretion time and Apgar score. Therefore, more research in these areas is still necessary. Finally, it is worth mentioning that the concentration and dose of carbohydrates in the studies were inconsistent, leading to significant heterogeneity. As a result, the determination of the optimal concentration and dose remains uncertain.

## 5. Conclusion

In conclusion, preoperative CHO intake alleviated patient discomfort as assessed by thirst, hunger, incidence of vomiting and nausea, insulin resistance, and time to flatus. Due to the low cost and convenience of CHO, preoperative CHO is feasible as a strategy to improve postoperative recovery. However, for the few studies, whether it is used as a routine operation of cesarean section still needs further research.

## Figures and Tables

**Table 1:** Details of included studies.

Authors, year	Country	Anesthesia	Carbohydrate group		Control group				Outcomes
					Placebo group		Fasting group		
			Sample size	Intervention	Sample size	Intervention	Sample size	Intervention	
Ghorashi et al. 2014 [18]	Iran	General anesthesia	267	Oral 150 ml of 10% traditional sugar plum 1 h preoperatively	—	—	144	Received nothing	Rate of regurgitation and aspiration
Razieh Khalooei Fard, 2017	Iran	Spinal anesthesia	45	Oral 800 ml of 12.5% carbohydrates (50 kcal/100 ml) between 20 and 24 hours before surgery and 400 ml of the same drink 2 hours before the anesthesia	46	Oral 800 ml of flavored water with lemon (0 kcal/100 ml) between 20 and 24 hours before surgery and 400 ml of the same drink 2 hours before the anesthesia	—	—	Time to first breastfeeding after surgery, frequency, and duration of breastfeeding for up to 36 hours after the surgery
Wendling et al. 2020 [23]	USA	Spinal anesthesia	15	Oral 710 mL of 14% carbohydrates (56.3 kcal/100 mL) the evening prior to surgery and 355 mL 2–4 hours before the anesthesia start time	17	Oral 710 mL of 5.9% carbohydrates (22.5 kcal/100 mL) the evening prior to surgery and 355 mL 2–4 hours before the anesthesia	15	Fasted after midnight the night before surgery and no oral intake at least 8 hours prior to surgery	Visual Analogue Scale (VAS) for hunger, thirst and well-being outcomes, blood loss, aspiration, breastfeeding, neonatal outcomes, and maternal complications
Clark, 2020	UK	Not mentioned	94	Oral 800 ml of 12% carbohydrate on the previous evening and another 400 ml 2 h prior to surgery. An extra 400 ml or further 400 ml 2 hours prior to surgery	90	Oral 800 ml of water on the previous evening and another 400 ml 2 h prior to surgery. An extra 400 ml or further 400 ml 2 hours prior to surgery	—	—	Urinary ketone level, dominant hand grip strength, anxiety score, hunger and thirst scores, and length of hospital stay
He et al. 2021 [19]	China	Spinal anesthesia	30	Oral 400 ml of 12.5% carbohydrate (50.9 kcal/100 mL) 2 hours before the surgery	29	Oral 400 ml clear water 2 hours before the surgery	29	Fasting for 10.5 hours	Postoperative insulin resistance, plasma glucose and insulin levels, VAS for thirst and hunger, the occurrences of nausea and vomiting, time of flatus, postoperative fever, Apgar score, and blood glucose level
Ningning Liu, 2020	China	Epidural anesthesia, a combination of epidural and lumbar anesthesia	43	Oral 300 mL 7.5% carbohydrate (30.5 kcal/100 mL) 2 hours before induction of anesthesia	42	Oral 300 mL warm water 2 hours before induction of anesthesia	—	—	Blood glucose, serum insulin, hunger and thirst scores, and gastrointestinal function parameters
Yuan Shi, 2020	China	Continuous epidural anesthesia	25	Oral 300 mL of 14.2% carbohydrate	25	Oral 300 mL of water	25	Fasting for 6 hours	VAS for thirst, hunger, and anxiety level, the gastric antral cross-sectional areas, insulin resistance, blood glucose, and insulin levels
Yang et al. 2021 [21]	China	Combined spinal-epidural anesthesia	39	Oral 300 mL of 14.1% carbohydrate (57.6 kcal/100 mL) at 2 hours before surgery	40	Oral 300 mL of distilled water at 2 hours before surgery	39	Fasting, drinking, or eating on the day of the operation	Core body temperature and body surface temperature, the incidence of intraoperative hypotension, vomiting, and shivering, amount of bleeding
Cheng et al. 2022 [15]	China	Combined spinal-epidural anesthesia	38	Oral 300 mL of 14.1% carbohydrate (57.6 kcal/100 mL) 2 h before the operation	37	Oral 300 mL of distilled water 2 h before the operation	37	Fasting, drinking, or eating on the day of the operation	The time to colostrum and exhaust, the incidence of vomiting, VAS of pain, vaginal bleeding volume, and complications (urinary retention, fever, or intestinal obstruction)

CHO, carbohydrate; VAS, Visual Analog Scale.

## Data Availability

No data were used to support this study.

## Supplementary Materials

Supplementary Materials Supplementary Appendix A: PubMed search strategy.
