# Supplementary material for: Effects of Preoperative Oral Carbohydrate on Perioperative Maternal Outcomes Undergoing Cesarean Section: A Systematic Review and Meta-Analysis
Source: Anesthesiol Res Pract. 2024 Mar 31;2024:4660422. doi: 10.1155/2024/4660422 (PMC10999288; doi:10.1155/2024/4660422)
Supplement: Supplementary Materials — Supplementary Appendix A: PubMed search strategy. [file 4660422.f1.doc]

**Supplementary Appendix A. Search strategy**

#1 Carbohydrates [Mesh]

#2 Dietary Carbohydrates [Mesh]

#3 carbs OR carbohydrate* [Title/Abstract]

#4 glucan* OR monosaccharide* OR disaccharide* OR oligosaccharide* OR polysaccharide* OR polycose OR lactose OR corn syrup [Title/Abstract]

#5 sucrose OR sugar* OR starch* OR saccharide* [Title/Abstract]

#6 #1 OR #2 OR #3 OR #4 OR #5

#7 Cesarean Section [Mesh]

#8 Cesarian OR cesarian section* [Title/Abstract]

#9 Caesarian OR Caesarean Section* OR caesarean [Title/Abstract]

#10 Cesarean Section* OR CS OR cesarean OR csection OR c section* [Title/Abstract]

#11 #7 OR #8 OR #9 OR #10

#12 randomized controlled trial OR controlled clinical trial [Publication Type]

#13 randomized OR placebo OR randomly OR trial [Title/Abstract]

#14 clinical trials as topic [Mesh]

#15 animals [Mesh] NOT humans [Mesh]

#16 #12 OR #13 OR #14

#17 #16 NOT #15

#18 #6 and #11 and #17
